# Three-Dimensional Calibration for Routine Analyses of Bromide and Nitrate Ions as Indicators of Groundwater Quality in Coastal Territories

**DOI:** 10.3390/ijerph16081419

**Published:** 2019-04-19

**Authors:** Francesco Parrino, Giovanni Camera-Roda, Vittorio Loddo, Leonardo Palmisano

**Affiliations:** 1 Department of Industrial Engineering, University of Trento, Via Sommarive 9, 38123 Trento, Italy; 2 Department of Civil, Chemical, Environmental, and Materials Engineering, University of Bologna, Via Terracini 28, 40131 Bologna, Italy; giovanni.cameraroda@unibo.it; 3 Department of Engineering (DI), University of Palermo, Viale delle Scienze Ed. 6, 90128 Palermo, Italy; vittorio.loddo@unipa.it (V.L.); leonardo.palmisano@unipa.it (L.P.)

**Keywords:** groundwater quality, bromide, nitrate, peak separation, poor resolved peaks, 3D calibration

## Abstract

Nitrate and bromide ions are generally considered indicators of anthropogenic pollution and seawater intrusion, respectively, in the groundwater of coastal territories. The analysis of these species is generally carried out with routine chromatographic analyses which generally afford partially merged or poorly resolved peaks. In the present paper a simple method for the correct evaluation of their concentration in water is reported. This method does not imply utilization of other instruments or technologies, only the mathematical elaboration of the data obtained from routine analysis of standard solutions containing the two species. Standard binary solutions of nitrate and bromide ions at different concentrations, ranging between 0.1 and 2 mM, were analyzed by means of ion chromatography. Splitting two partially merged chromatographic peaks and considering each resulting area as originating from a single species produces “measured” concentration values which differ from the nominal ones. Such a procedure generates errors (one per species) which can be written as a function of the above mentioned “measured” concentrations and which can be graphically represented by means of a surface in a three-dimensional (3D) space. In this way, “measured” concentrations of bromide and nitrate ions can be corrected by calculating the errors generated under the experimental conditions at which the chromatographic separation is performed. Notably, this is analogous with the two-dimensional (2D) calibration normally carried out for analytical purposes. Indeed, both methods allow estimation of the unknown concentration of species in solution by correlating the instrumental response with the concentration of standard solutions.

## 1. Introduction

The sustainable exploitation of water may be considered one of the greatest challenges of the current century [1]. Indeed, the global water crisis, both from qualitative and quantitative points of view, is strictly related to human activities and generally to environmental degradation. For these reasons, developing simple methods to test water quality is a fundamental prerequisite for assessing efficient management of the water resources [2,3]. Generally, both natural and anthropogenic contaminants may affect the water quality [4,5,6]. It is already of common awareness that non-sustainable agriculture, farming, or industrial activities cause direct contamination of surface water and/or groundwater. Nitrate ion levels can be considered a preliminary indicator of water quality [7]. Naturally occurring levels in the USA, for instance, generally do not exceed 4–9 mg·L^−1^ for nitrate and 0.3 mg·L^−1^ for nitrite in groundwater [8]. The 1991 European directive 91/676 [9] establishes that nitrate concentration in fresh water and groundwater must remain below 50 mg·L^−1^. However, as a result of agricultural activities, the nitrate concentration can easily reach several hundred milligrams per liter. For example, concentrations up to 1500 mg·L^−1^ were detected in groundwater of a cultivated area in India [10]. High nitrate concentrations reflect water pollution deriving from fertilizers, manures, food processing, munitions, and some polyresin facilities [11]. It has been demonstrated that consumption of water with higher amounts of nitrates results in serious health problems for human beings [12,13]. Infant methemoglobinemia is classically associated with the presence of nitrate in water and the regulation limits have been mainly set according to the related epidemiologic studies. However, many recent studies have observed increasing risks related to the presence of nitrate in drinking water at levels below the regulatory limits. These risks include colorectal, bladder, and breast cancer; reproductive problems; neural tube defects; and thyroid diseases [14].

On the other hand, it is less known that the indiscriminate utilization of groundwater in coastal territories generates seawater intrusion resulting in serious water quality degradation. Bromide ion may serve as a useful tracer of this occurrence [15]. In general, bromide ion is present at concentrations lower than 0.1 mg·L^−1^ in nearly all drinking water [16], but seawater intrusions highly increase its concentration in groundwater. Moreover, the release of bromine into the environment from anthropogenic activities can also increase its amount in groundwater up to three times [17]. Human-derived bromide sources are mining, emission of brominated compounds used as scavenger in leaded fuels [18], flame retardants, fertilizers, and pesticides [19]. Bromide is not harmful per se, but it can take part in reactions producing carcinogenic brominated compounds and bromine under natural conditions. Moreover, in contaminated water effluents, the presence of bromide ions strongly influences the choice of the treatment method [20,21,22,23]. Indeed, ozone-based purification processes afford fast degradation of the organic pollutants but they also oxidize, almost quantitatively, bromide to bromate ions, or in some case to bromine [24,25], both of which are toxic and carcinogenic species even at very low concentrations [26].

Moreover, it has been demonstrated that the ratio (R) between the concentration of chloride and bromide in groundwater is a powerful indicator not only of seawater intrusion but also of pollution due to anthropogenic activities [27]. In fact, the physical processes which occur in the soil affect the absolute concentrations of the ions but do not modify their ratio. Therefore, due to the high solubility and small ionic size of these ions, the ratio (R) keeps memory of the natural or anthropogenic changes of the groundwater composition. Notably, while the determination of the chloride concentration is generally straightforward, the determination of bromide ions presents analytical issues as discussed below.

For these reasons, the simultaneous determination of bromide and nitrate ions is of paramount importance in preliminary water quality tests in order to identify the source(s) of pollution of the soil and of groundwater. Ion chromatography is a powerful tool for detection of bromide and nitrate ions [28,29,30,31]. The ions are separated by means of an ion exchange column and detected through a conductivity detector. However, this method allows exact quantification only when the resolution of the peaks is good, but the use of low-efficiency anion exchange columns may generate significant errors in figures deriving from analyses of real water matrices. Bromide and nitrate peaks generally appear close to each other, often asymmetrical and partially merged. In this case, being the area below the peaks related to both species, it is impossible to determine the real contribution of each peak by simply splitting them through a vertical line or just using different baselines. In fact, the area of each peak will be influenced by the extent of the overlap. Notably, the problem of separating slightly merged peaks has been often underestimated. Indeed, the operation of “peak splitting” is available in every chromatography software package and it is often performed without considering the high errors generated, which in some cases may reach even 80%. Changing some eluent related parameters does not result in significant improvements. In fact, only the retention time of the ions will be affected, while the selectivity is satisfactory only by using high-efficiency columns [32]. It is worth noting that dilution of the samples would improve the separation only if the two species were present in similar and low concentration, which is rarely the case when the above-mentioned typical concentrations of bromide and nitrate in polluted groundwater are taken into account. Colorimetric methods are often time-demanding and may suffer interferences from other species possibly present in the sample [33,34]. Deconvolution and computer-assisted methods [35] are generally not easily available for routine measurements and generally fail for real wastewater applications [36]. Using high-efficiency ion exchanging columns and ultraviolet-visible (UV-vis) or conductivity detectors may solve the problem [37,38,39,40,41]. However, these methods often suffer interference by high nitrate concentrations (>100 mg·L^−1^) [38]. Electrochemical detectors may be used for determination of bromide in aqueous samples [42,43,44,45,46] but not for nitrate ions, which do not produce any signal. Electrostatic ion chromatography [47] and capillary zone electrophoresis [48] have been also used to separate bromide and nitrate ions in complex matrices but these apparatuses are not generally available for routine analyses. UV absorbance detectors are not selective for bromide and nitrate ions. Interestingly, Tirumalesh reported a combination of UV and amperometric detection for the simultaneous determination of bromide and nitrate using low-efficiency anion exchange columns [36].

The present paper proposes a more heuristic approach to the problem. The discrepancy between the concentration measured by splitting the chromatographic peaks and the nominal concentration was calculated for standard samples and its dependence on the measured concentrations has been modelled. The three-dimensional (3D) surface obtained in this way can be used for a more accurate estimation of the concentration in aqueous matrices. We are aware that chromatographic systems of new generation, peak modelling or deconvolution methods may be an efficient alternative to this method. However, often old instruments with poor resolution are used for routine analysis and the current economic crisis dramatically limits the financial possibilities of many research centers which must continue working with the available instruments. Moreover, most of the old chromatography software packages do not produce digitalized chromatograms, so that peak modelling procedures [49,50] and deconvolution methods are not straightforward. The present method is simple and reliable and is based on a calibration procedure applied on mixtures of two components.

## 2. Experimental

All of the analyses were conducted with a Dionex ion chromatography equipment (DX-120, Dionex, Sunnyvale, CA, USA) consisting of a pump, a Rheodyne injection port, an anion guard column (Ionpac AG14A, 4 × 50 mm Guard), an anion separating column (Ionpac AS14A, 4 × 250 mm Analytical), an anion self-regenerating suppressor unit (ASR5-300, 4 mm) and a conducibility detector (CDM-3). A Dionex Peaknet (version 5.01) chromatography workstation was used for system control and data collection. For the determination of bromide, nitrate, and chloride ions, the mobile phase was a 1–8 or 1–3.2 mM NaHCO_3_-Na_2_CO_3_ buffer solution, with a flow rate of 1 mL·min^−1^. The mobile phase at 1–8 mM produced retention times of chloride, bromide, and nitrate of 3.67, 5.22, and 5.78 min, respectively. When the NaHCO_3_-Na_2_CO_3_ concentration was 1–3.2 mM, the retention times of the above mentioned ions were 5.15, 7.35, and 8.18 min, respectively. The injection volume was 25 µL and the column backpressure was ca. 1900 psi. The bromide and nitrate concentrations used in this work ranged between 0.1 and 2 mM.

All the chemicals used were of analytical grade quality (Sigma-Aldrich). Standard solutions and the mobile phase were prepared by using deionized water (conducibility ≤ 0.08 µS).

Before starting this work, the chromatographic apparatus was tested according to the procedure described in the operative manual. In particular, the conductivity test was performed by injecting a standard (10 mg·L^−1^) HNO_3_ solution, provided by Dionex, into the detection cell. The signal which was obtained indicated a performing cell detector. The stationary phase was purified prior to use, according to the procedure described by the operative manual. In particular, the column was washed for 60 min with a solution containing 200 mM HCl in water and acetonitrile (20:80 *v/v*), rinsed with water, washed with an aqueous solution 80 and 10 mM of Na_2_CO_3_ and NaHCO_3_, respectively, and again rinsed with water. After this procedure the column performance was evaluated by comparing the nominal peak resolutions with those obtained by injecting a standard solution containing fixed amounts of fluoride, acetate, chloride, nitrite, bromide, nitrate, phosphate, and sulfate ions. The peak resolutions obtained were ca. 68% with respect to the nominal ones, indicating that the column performances were ca. 30% lower with respect to the optimum conditions.

Fitting of the experimental data was performed by means of Origin 8.5 platform (OriginLab, Northampton, MA, USA). Analyses were repeated several times in order to test the repeatability of the chromatographic response. In particular, for bromide and nitrate ions the standard deviation calculated on five different analyses of the same sample ranged between 0.004 and 0.01, respectively.

## 3. Results and Discussion

**Figure 1:**
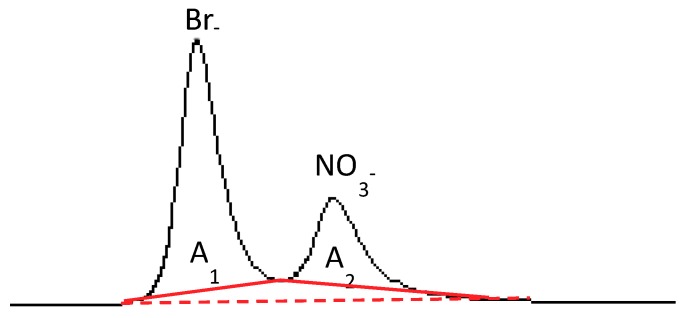
Integration carried out by considering two different baselines.

Injecting an aqueous solution containing bromide (2 mM) and nitrate (1 mM) ions produced the chromatogram depicted in Figure 1. Also shown is one of the different ways to integrate the peaks, (i.e., by considering two different baselines).

The bromide and nitrate peaks are partially merged and show poor resolution as described in the experimental part. The evaluation of the areas A_1_ and A_2_ shown in Figure 1 is prone to quantification errors. Attempts to separate the peaks by changing the eluent concentration failed as this parameter scarcely influences the selectivity of the peaks, strongly affecting only the retention of the ions. Indeed, the area A_1_ results not only from the contribution of bromide ions eluting through the column but also from the nitrate ions eluted in the same time. The same considerations can be made for the second peak. Therefore, simple conversion of A_1_ and A_2_ into concentration values by means of a calibration curve of the single ions gives rise to not negligible errors. The approach proposed in this paper is based on the consideration that the error generated by means of this procedure is a function of the two areas, A_1_ and A_2_, which are regarded as independent parameters. A_1_ and A_2_ have been converted into concentration values through a normal calibration with the standard solution containing only one species because the resulting area values are less meaningful than the concentration values. The obtained concentration values will be labelled as “measured” (Meas.) values, indicating that A_1_ and A_2_ are considered as being generated only from bromide and nitrate ions, respectively, neglecting the overlapped area. In order to convert A_1_ and A_2_ in Meas.[Br^−^] and Meas.[NO_3_^−^], respectively, two calibrations were performed—the first by using five standard aqueous solutions containing only bromide and the second by using five standard solutions containing only nitrate ions. The coefficient of determination (*R*^2^) and the slope of the calibration of straight lines obtained are reported in Table 1. Data are reported for NaHCO_3_-Na_2_CO_3_ eluent concentrations of 1 mM–8 mM and 1 mM–3.2 mM. Notably, the response of the ion chromatograph was often checked with nitrate and bromide standard solutions purchased from Merck.

In this way the error generated by the merging of peaks will be a function of Meas.[Br^−^] and of Meas.[NO_3_^−^]. The discrepancy between Meas.[Br^−^] and the nominal [Br^−^] (Nom.[Br^−^]) may be described by defining the parameter E[Br^−^] as it follows:

E[Br^−^] = {(Nom.[Br^−^] − Meas.[Br^−^])/Nom.[Br^−^]} × 100. (1)

E[NO_3_^−^] can be analogously defined:

E[NO_3_^−^] = {(Nom.[NO_3_^−^] − Meas.[NO_3_^−^])/Nom.[NO_3_^−^]} × 100. (2)

E[Br^−^] and E[NO_3_^−^] indicate how different the real concentration of bromide (Nom.[Br^−^]) and of nitrate (Nom.[NO_3_^−^]) are from Meas.[Br^−^] and Meas.[NO_3_^−^], respectively. E[Br^−^] and E[NO_3_^−^] depend on both A_1_ and A_2_ (i.e., on both Meas.[Br^−^] and Meas.[NO_3_^−^]) as summarized by Equations (3) and (4).

E[Br^−^] = *f* (Meas.[Br^−^], Meas.[NO_3_^−^]) (3)

E[NO_3_^−^] = *g* (Meas.[Br^−^], Meas.[NO_3_^−^]) (4)

As previously described, Meas.[Br^−^] and Meas.[NO_3_^−^] values can be easily obtained for all of the samples containing unknown amounts of nitrate and bromide. Therefore, once known, *f* and *g* allow estimation of the nominal concentration of bromide and nitrate, respectively, by correcting their “measured” concentrations. However, a valid and general expression of Equations (3) and (4) does not exist, because *f* and *g* functions strictly depend on the experimental conditions of the chromatographic separation. Indeed, E[Br^−^] and E[NO_3_^−^] depend on the shape, height, resolution, area (integration method), and mutual distance of the peaks which change by changing the chromatographic conditions. However, once these parameters are fixed, *f* and *g* may be univocally identified.

In order to find the expression of *f* and *g,* we used a procedure similar to the usual calibration performed to quantify a single species. Different standard solutions containing both nitrate and bromide ions in various proportions and at nominal concentrations ranging from 0.1 to 2 mM were prepared and analyzed by means of ion chromatography, using 1–3.2 mM and 1–8 mM NaHCO_3_-Na_2_CO_3_ eluents. The obtained chromatographic peaks were integrated as shown in Figure 1 and Meas.[Br^−^] and Meas.[NO_3_^−^] values were obtained for each solution. Being the nominal amount of bromide (Nom.[Br^−^]) and nitrate (Nom.[NO_3_^−^]) known, it is possible to calculate E[Br^−^] and E[NO_3_^−^] for each solution according to Equations (1) and (2).

In order to show the dependence of E[Br^−^] and E[NO_3_^−^] on Meas.[Br^−^] and Meas.[NO_3_^−^] in the clearest way, it is opportune to fix the concentration of one of the two species so that the trend can be visualized in a two-dimensional plot. The results hereby reported have been obtained by using the integration method shown in Figure 1 and the eluent 1–3.2 mM.

**Figure 2:**
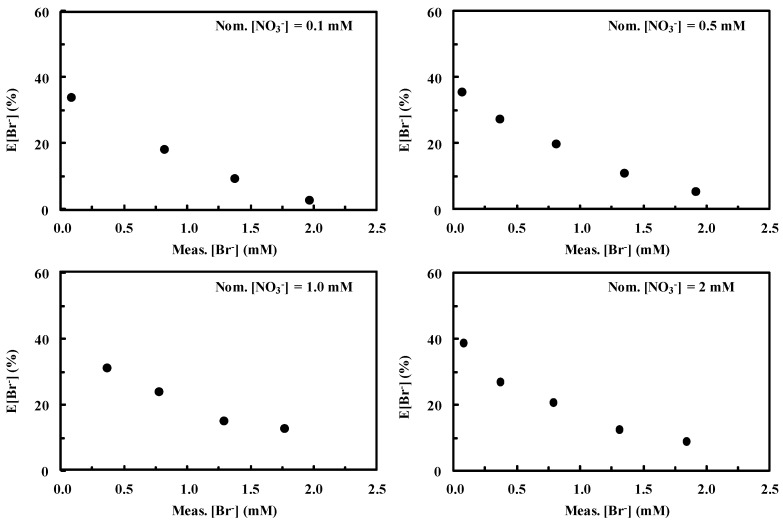
Dependence of E[Br^−^], defined according to Equation (1), on measured [Br^−^] (Meas.[Br^−^]) at fixed nitrate concentration. Eluent 1 mM NaHCO_3_–3.2 mM Na_2_CO_3_. Figures were obtained by integrating the peaks according to Figure 1.

Figure 2 shows the dependence of E[Br^−^] on the Meas.[Br^−^] at constant Nom.[NO_3_^−^]. It is evident that the amount of nitrate virtually does not influence E[Br^−^] in the concentration range considered. On the contrary, increasing Meas.[Br^−^] values results in decreasing E[Br^−^]. Indeed, as the second peak virtually does not influence the error generated in the first one, the latter results lower for higher bromide concentrations. In particular, E[Br^−^] ranges from values of ca. 40% for low Meas.[Br^−^] to values lower than 10% for higher Meas.[Br^−^].

**Figure 3:**
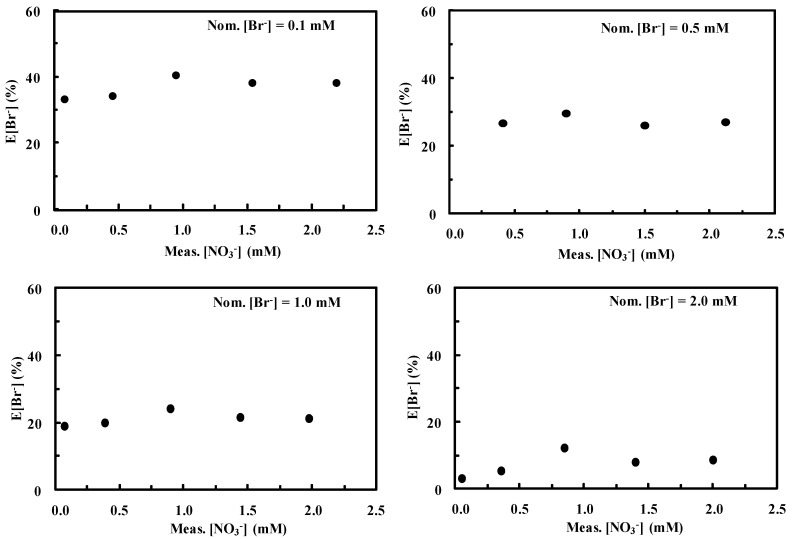
Dependence of E[Br^−^] on measured [NO_3_^−^] (Meas.[NO_3_^−^]) at fixed bromide concentration. Eluent 1 mM NaHCO_3_–3.2 mM Na_2_CO_3_. Figures were obtained by integrating the peaks according to Figure 1.

Figure 3 shows the dependence of E[Br^−^] on Meas.[NO_3_^−^] at fixed bromide concentration.

Variations of Meas.[NO_3_^−^] did not virtually produce changes in E[Br^−^]. On the other hand, higher Nom.[Br^−^] values produced lower errors in evaluating bromide concentration. In particular E[Br^−^] ranges from ca. 40 to less than 10% for Nom.[Br^−^] values ranging from 0.1 to 2 mM, as also observed in Figure 2.

Figure 2 and Figure 3 clearly demonstrate the existence of a trend in the error generated by integrating peaks according to Figure 1. This trend may be mathematically expressed through Equation (3) by means of a nonlinear best fitting procedure in a 3D space where Meas.[NO_3_^−^], Meas.[Br^−^], and E[Br^−^] are reported as the x, y, and z axes, respectively. Among the possible fitting functions, polynomials in the form

(5) $$E\left[{\mathrm{Br}}^{-}\right]=a_{0}+\sum_{i=1}^{m}a_{i}\times {\left(\mathrm{Meas}.\left[{\mathrm{Br}}^{-}\right]\right)}^{i}+\sum_{j=1}^{n}a_{j}\times {\left(\mathrm{Meas}.\left[{\mathrm{NO}}_{3}^{-}\right]\right)}^{j}$$

best describe the trend of the experimental points.

Notably, the higher the degree of the polynomial, the better points are fitted by the function whose coefficients of determination result closer to one. However, this spurious enhancement of the coefficient of determination does not correspond to a better choice for the purposes of the present method. Indeed, it is necessary to consider the adjusted coefficient of determinations (Adj.*R*^2^) in order to avoid over-parameterization of the model. On the other hand, it is not required that the fitting surface exactly matches all of the experimental points, but it must describe their trend. In order to mediate between these two factors one can consider the Adj.*R*^2^ values for degrees of the polynomial functions (*m* and *n*) ranging from 2 to 5 (see Table 2).

Although very good fitting can be achieved with *m* = *n* = 5, the resulting surface does not consider the continuity of the observed trend, strongly accounting for possible fluctuation of the experimental points. Conversely, the polynomial with *m* = *n* = 2 can be considered a better compromise for the aim of this work as an acceptable Adj.R^2^ value is obtained for a surface with a good description capability of the trend of E[Br^−^].

To sum up, the experimental points can be fitted by the following Equation:

Er[Br^−^] = Z + A·(Meas.[NO_3_^−^]) + B·Meas.[Br^−^] + C·(Meas.[NO_3_^−^])^2^ + D·(Meas.[Br^−^])^2^. (6)

The values of the parameters in Equation (6) and Adj.*R*^2^ values are listed in Table 3.

**Figure 4:**
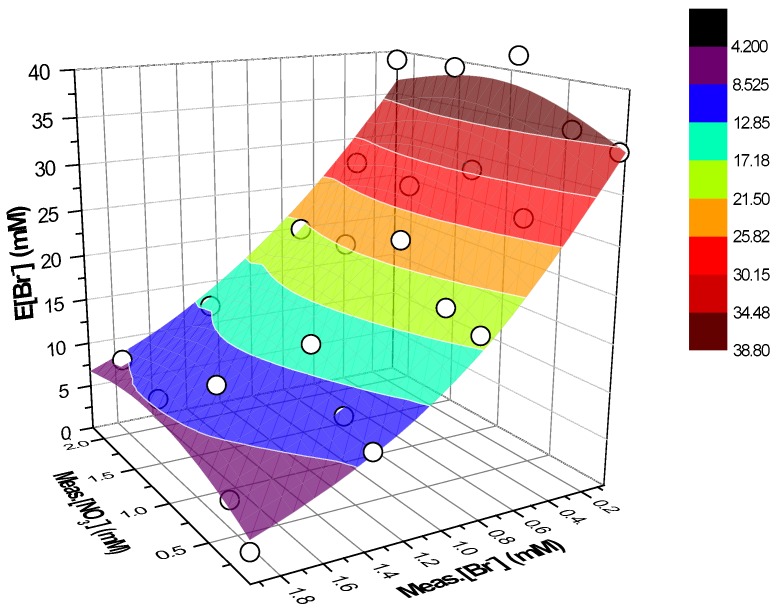
Three-dimensional (3D) surface fitting the experimental data (white points) as triads (x, y, z) = (Meas.[NO_3_^−^], Meas.[Br^−^], E[Br^−^]). Eluent 1 mM NaHCO_3_–3.2 mM Na_2_CO_3_. Integration of the peaks as shown in Figure 1.

Equation (6) describes the surface reported in Figure 4 along with the experimental values represented through white circles.

**Figure 5:**
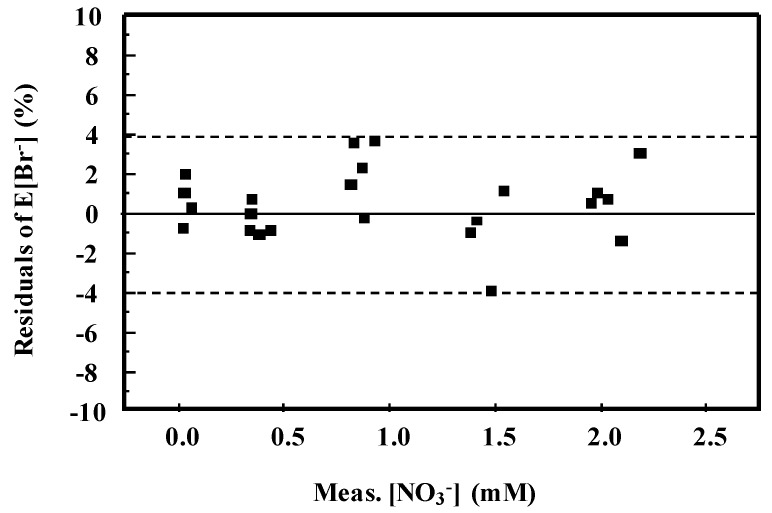
Residuals of E[Br^−^] (difference between experimental points and fitted values) vs. Meas.[NO_3_^−^].

The difference between the surface and the experimental points, expressed as residuals of E[Br^−^], ranges between ±4% showing a good data correlation (Figure 5).

A similar approach can be used to evaluate the dependence of E[NO_3_^−^] on Meas.[Br^−^] at fixed nominal concentration of nitrate and on Meas.[NO_3_^−^] at fixed nominal values of bromide.

Plotting the experimental values in a 3D space with the x, y, and z axes being Meas.[NO_3_^−^], Meas.[Br^−^], and E[NO_3_^−^], respectively, affords points which may be non-linearly fitted by means of Origin 8.5 platform. In this case, a polynomial function in the form expressed by Equation (5) may also be one of the best options. Considerations similar to the case of E[Br^−^] can be made, so that the polynomial function with *m* = *n* = 2, expressed by Equation (7), can be chosen as the best compromise between a good fitting and a trend description ability.

Er[NO_3_^−^] = Z + A·(Meas.[NO_3_^−^]) + B·Meas.[Br^−^] + C·(Meas.[NO_3_^−^])^2^ + D·(Meas.[Br^−^])^2^ (7)

The values of the parameters in Equation (7) and the Adj.*R*^2^ value are listed in Table 4.

**Figure 6:**
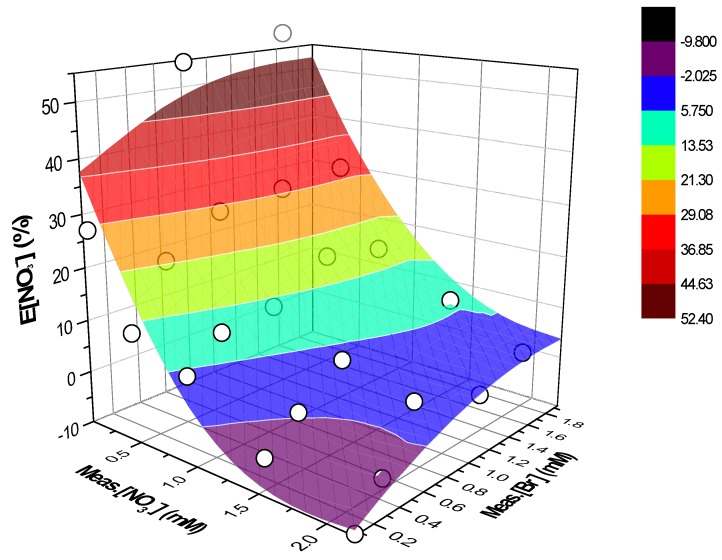
3D surface fitting the experimental data (white points) as triads (x, y, z) = (Meas.[NO_3_^−^], Meas.[Br^−^], E[NO_3_^−^]). Eluent 1 mM NaHCO_3_–3.2 mM Na_2_CO_3_. Integration of the peaks as shown in Figure 1.

Equation (7) describes the surface reported in Figure 6 along with the experimental values represented through white circles.

The difference between the surface and the experimental points, expressed as residuals of E[NO_3_^−^], ranges between ±5%.

**Figure 7:**
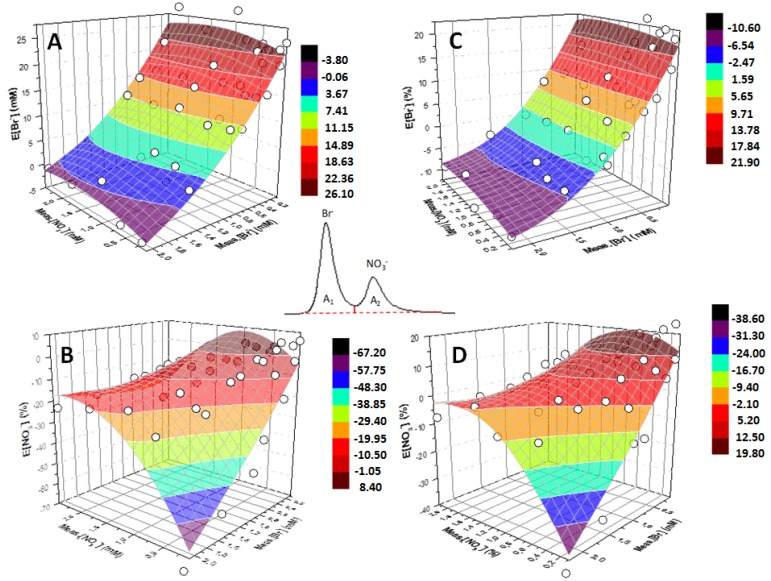
Inset: Chromatographic peaks obtained for a solution containing bromide (2 mM) and nitrate (1 mM) ions, integrated by splitting them with the same baseline. The integration method depicted in the inset has been used to retrieve the 3D surfaces fitting the experimental data (white circles) as triads (x, y, z) = (Meas.[NO_3_^−^], Meas.[Br^−^], E[Br^−^]) and (Meas.[NO_3_^−^], Meas.[Br^−^], E[NO_3_^−^]), for Figure 7A–C and B–D, respectively. Figure 7A,B are obtained by using eluent 1 mM NaHCO_3_–3.2 mM Na_2_CO_3_, while Figure 7C,D are obtained by using eluent 1 mM NaHCO_3_–8 mM Na_2_CO_3_.

In order to test the reliability and general validity of the method, the influence of a different integration method and of a different eluent concentration was investigated following the same approach. For the sake of brevity, only the 3D surfaces described by means of polynomial functions are reported in Figure 7. The experimental data have been retrieved by integrating the chromatographic peaks with the same baseline (see inset within the plots of Figure 7). In particular, Figure 7A,B express the dependence on Meas.[NO_3_^−^] and Meas.[Br^−^] of E[Br^−^] and E[NO_3_^−^], respectively, by using eluent 1 mM NaHCO_3_–3.2 mM Na_2_CO_3_. Analogously, Figure 7C,D are obtained by using eluent 1 mM NaHCO_3_–8 mM Na_2_CO_3_.

Changing the integration method results in differences in the mutual contribution of the two signals producing slightly different surfaces. On the other hand, changing the eluent composition produces surfaces with similar shapes but with different coordinates. Indeed, changing the retention time and the distance between the two peaks results in differences in merging and mutual contribution of the two signals. However, these changes do not affect the validity of the method, as in any case it still exists a function relating E[NO_3_^−^] and E[Br^−^] with Meas.[Br^−^] and Meas.[NO_3_^−^]. Finally, it is worth mentioning that the presence of chloride ions did not influence either retention times or the area A_1_ and A_2_ of the nitrate and bromide peaks, respectively, so the method can still be successfully used.

## 4. Conclusions

Routine analyses of nitrate and bromide ions are essential for evaluating the quality of groundwater and of the soil. In fact, nitrate concentrations higher than ca. 10 mg∙L^−1^ reflect water pollution possibly deriving from fertilizers, manures, food processing, munitions, and some polyresin facilities, while bromide concentration higher than ca. 0.1 mg∙L^−1^ may be related to emission of brominated compounds, flame retardants, fertilizers, and pesticides. These ions are not only tracers of environmental pollution but, in particular, nitrate ions are harmful above certain concentrations and bromide ions may induce formation of toxic brominated compounds under natural conditions. For these reasons, chromatographic analyses are routinely performed to detect these ions in groundwater. Unfortunately, poorly resolved and partially merged chromatographic peaks are obtained with the instruments generally available. The method proposed in this paper allows correct evaluation of the concentration of bromide and nitrate ions without the need of other (efficient but expensive) techniques. Integrating two partially merged chromatographic peaks and considering the resulting areas as originated by the single species produces “measured” concentrations of the two species which differ from the nominal concentrations. Such discrepancy generates an error in the quantification of the two species. The present work shows that this error is a function of the “measured” concentrations of the two species. In particular, this dependence can be mathematically expressed by means of a 3D surface fitting the experimental data. Such procedure is similar to the normal calibration performed for analytical quantification of single species. Indeed, both methods allow estimation of the unknown concentration of species in solution, by correlating the instrumental response with the concentration of standard solutions. In fact, once an unknown sample is analyzed in the same experimental conditions of the 3D calibration, two “measured” concentration values can be easily obtained and, after substituting these values in the equation of the 3D surface, a correction factor can be derived in order to obtain a more accurate quantitative evaluation of the species. This method does not imply utilization of other instruments or technologies but only the mathematical elaboration of the data obtained from the analysis of standard solutions containing the two species. The method was applied to solutions containing bromide and nitrate ions whose chromatographic peaks are partially merged, but can be extended to all the cases where species with poorly resolved or overlapping peaks must be quantitatively determined. 

## Figures and Tables

**Table 1:** Slopes and coefficients of determination obtained for the calibration lines of bromide and nitrate at NaHCO_3_/Na_2_CO_3_ eluent concentration of 1 mM–8 mM and 1 mM–3.2 mM.

Ions	Eluent 1–3.2 mM		Eluent 1–8 mM	
	Slope	*R* ^2^	Slope	*R* ^2^
**Bromide**	0.585	0.992	0.672	0.999
**Nitrate**	0.651	0.993	0.575	0.997

**Table 2:** Adjusted coefficients of determination (Adj.*R*^2^) values for different polynomials fitting E[Br^−^] values, with the degree of x = Meas.[NO_3_^−^] (*m*) and of y = Meas.[Br^−^] (*n*) listed in the first column and the first row, respectively.

	*n* = 2	*n* = 3	*n* = 4	*n* = 5
*m* = 2	0.971	0.978	0.979	0.983
*m* = 3	0.973	0.978	0.980	0.984
*m* = 4	0.979	0.983	0.985	0.991
*m* = 5	0.980	0.984	0.987	0.993

**Table 3:** Parameters Z, A, B, C, D and adjusted coefficient of determination (Adj.*R*^2^) for the fitting curve described by Equation (6).

**Z**	35.438	**C**	−2.962
**A**	7.738	**D**	6.016
**B**	−27.897	**Adj.*R*^2^**	0.973

**Table 4:** Parameters Z, A, B, C, D and adjusted coefficient of determination (Adj.*R*^2^) for the fitting curve described by Equation (7).

**Z**	38.553	**C**	15.328
**A**	−55.776	**D**	−5.661
**B**	19.073	**Adj.*R*^2^**	0.903
